# Genetic polymorphism in the manganese superoxide dismutase gene, antioxidant intake, and breast cancer risk: results from the Shanghai Breast Cancer Study

**DOI:** 10.1186/bcr929

**Published:** 2004-09-22

**Authors:** Qiuyin Cai, Xiao-Ou Shu, Wanqing Wen, Jia-Rong Cheng, Qi Dai, Yu-Tang Gao, Wei Zheng

**Affiliations:** 1 Department of Medicine and Vanderbilt–Ingram Cancer Center, Vanderbilt University, Nashville, Tennessee, USA; 2 Department of Epidemiology, Shanghai Cancer Institute, Shanghai, China

**Keywords:** antioxidant, breast cancer, case-control study, MnSOD, polymorphism

## Abstract

**Introduction:**

It has been suggested that oxidative stress and mitochondrial DNA damage play important roles in breast cancer carcinogenesis. Manganese superoxide dismutase (MnSOD) is a major enzyme that is responsible for the detoxification of reactive oxygen species in the mitochondria. A T → C substitution in the *MnSOD *gene results in a Val → Ala change at the -9 position of the mitochondrial targeting sequence (Val-9Ala), which alters the protein secondary structure and thus affects transport of MnSOD into the mitochondria.

**Methods:**

We evaluated this genetic polymorphism in association with breast cancer risk using data from the Shanghai Breast Cancer Study, a population-based case–control study conducted in urban Shanghai from 1996 to 1998. The *MnSOD *Val-9Ala polymorphism was examined in 1125 breast cancer cases and 1197 age-frequency-matched control individual.

**Results:**

Breast cancer risk was slightly elevated in women with Ala/Ala genotype (odds ratio [OR] 1.3, 95% confidence interval [CI] 0.7–2.3), particularly among premenopausal women (OR 1.8, 95% CI 0.9–3.7), as compared with those with Val/Val genotype. The increased risk with the Ala/Ala genotype was stronger among premenopausal women with a higher body mass index (OR 2.5, 95% CI 0.9–7.0) and more years of menstruation (OR 2.6, 95% CI 0.8–8.0). The risk among premenopausal women was further increased twofold to threefold among those with a low intake of fruits, vegetables, vitamin supplements, selenium, or antioxidant vitamins, including carotenes and vitamins A, C, and E. However, the frequency of the Ala allele was low (14%) in the study population, and most of the ORs provided above were not statistically significant.

**Conclusion:**

The present study provides some evidence that genetic polymorphism in the *MnSOD *gene may be associated with increased risk of breast cancer among Chinese women with high levels of oxidative stress or low intake of antioxidants. Studies with a larger sample size are needed to confirm the findings.

## Introduction

More than 90% of the body's oxygen is consumed by the electron transport chain in mitochondria [1], and about 1–5% of it is released as superoxide (O_2_^•-^) and hydrogen peroxide (H_2_O_2_) [2]. Reactive oxygen species (ROS) may also be generated from estrogen metabolism through catechol estrogen redox cycling [3,4]. Because of a high level of internally generated ROS, lack of histone protection, and a low level of DNA repair, mitochondrial DNA is particularly vulnerable to oxidative damage [5]. It has been suggested that mitochondrial DNA damage may play an important role in breast carcinogenesis [5,6]. Manganese superoxide dismutase (MnSOD) and glutathione peroxidase (GPX) 1 are two major enzymes that are responsible for ROS detoxification in mitochondria [7,8]. The *MnSOD *gene, which is composed of five exons and four introns, is localized to chromosome 6q25 [9,10]. A T → C substitution, resulting in a Val → Ala change at the -9 position (Val-9Ala), which alters the secondary structure of the protein [11], has been noted to affect transport of MnSOD into the mitochondria [12,13]. Another polymorphism (Ile58Thr) in exon 3 affects the stability of MnSOD and reduces protein amount and enzyme activity [14,15]. Cells that over-expressed the Ile58 allele had higher MnSOD activity than did cells that over-expressed the Thr58 allele [16]. It is biologically plausible that the Val-9Ala and Ile58Thr polymorphisms play an important role in ROS detoxification, thus affecting the risk for developing cancer, particularly among individuals with a higher level of oxidative stress or who are deprived of other antioxidative protection, such as through a low level of antioxidant intake.

Four studies examined the association of the Val-9Ala polymorphism with breast cancer risk, with mixed results [17-20]. Two hospital-based case–control studies found a moderate elevation in risk among women carrying the Ala/Ala genotype [17,18]. A population-based case–control study found no overall association with this polymorphism [19]. Recently, a case–control study conducted using data from the Ontario Familial Breast Cancer Registry found no association with this polymorphism [20]. All four studies were conducted among Caucasian women and had relatively small sample sizes, and most of them did not include a comprehensive assessment of environmental exposures or lifestyle data. Using data from the Shanghai Breast Cancer Study, a large-scale population-based case–control study conducted in urban Shanghai from 1996 to 1998, we evaluated the association of the *MnSOD *gene polymorphism with breast cancer risk, in conjunction with conditions related to oxidative stress and dietary intake of antioxidants.

## Methods

### Study participants

The cases and control individuals evaluated in this study were participants of the Shanghai Breast Cancer Study, a population-based case–control study. Detailed study methods were published elsewhere [21]. Briefly, the study included 1459 women aged between 25 and 64 years, who were diagnosed with breast cancer between August 1996 and March 1998, and 1556 age-frequency-matched control individuals. The study protocol was approved by committees of all relevant institutions for the study of humans in research. All study participants were permanent residents of urban Shanghai who had no prior history of cancer and were alive at the time of interview. Through a rapid case ascertainment system, supplemented by the population-based Shanghai Cancer Registry, a total of 1602 eligible patients with breast cancer were identified during the study period, and interviews were completed in-person by 1459 (91%) of them. The major reasons for nonparticipation were refusal (109 [6.8%]), death before the interview (17 [1.1%]), and inability to locate the person (17 [1.1%]). Cancer diagnoses for all patients were confirmed by two senior study pathologists by review of tumor slides.

Control individuals were selected using the Shanghai Resident Registry, a population registry containing demographic information for all residents of urban Shanghai, and were frequency matched for age (5-year intervals) to the expected age distribution of the cases in a 1:1 ratio. The inclusion criteria for control individuals were identical to those for the cases but with the exception of a diagnosis of breast cancer. Of the 1724 eligible women, 1556 (90.3%) completed interviews in-person. The remaining women were not included in the study either because of refusal (166 [9.6%]) or death before the interview (2 [0.1%]).

A structured questionnaire was used to elicit detailed information on demographic factors, menstrual and reproductive histories, hormone use, dietary habits, prior disease history, physical activity, tobacco and alcohol use, weight, and family history of cancer. For all participants, current weight, circumference of the waist and hips, and height while sitting and standing were measured. Blood samples were obtained from 1193 (82%) cases and 1310 (84%) control individuals who completed in-person interviews. These samples were processed on the same day, typically within 6 hours of sample collection, and stored at -70°C until bioassays were performed.

Usual dietary habits over the past 5 years were assessed by in-person interview, using a validated quantitative food frequency questionnaire [22,23]. The food frequency questionnaire included 76 food items or groups, 30 fresh vegetables and nine fruits, covering over 85% of foods commonly consumed in Shanghai. During the in-person interview, each study participant was first asked how frequently she consumed a specific food or group of foods (daily, weekly, monthly, yearly, or never), followed by a question on how many liangs (= 50 g) of food were eaten per unit time (day, week, month, or year) during the previous 5-year period, ignoring any recent changes in usual dietary intake within the 5-year period. Total dietary intakes of vitamin A (mg), carotene (mg), vitamin C (mg), vitamin E (mg), and selenium (μg) were calculated based on data from the Chinese Food Composition Table [24].

### Genotyping method

Genomic DNA was extracted from buffy coat fractions. The *MnSOD *genotypes were determined with PCR–restriction fragment length polymorphism methods, as reported previously [17,25] but with some modification. Briefly, the primers for the Val-9Ala polymorphism were 5'-ACCAGCAGGCAGCTGGCGCCGG-3' (forward) and 5'-GCGTTGATGTGAGGTTCCAG-3' (reverse). The primers for the *Ile58Thr *polymorphism were 5'-AGCTGGTCCCATTATCTAATAG-3' (forward) and 5'-TCAGTGCAGGCTGAAGAGAT-3' (reverse). The PCR was performed in a PTC-200 Peltier Thermal Cycler (MJ Research Inc., Waltham, MA, USA). Each 20 μl of PCR mixture contained 5 ng DNA, 1× PCR buffer with 1.5 mmol/l MgCl_2_, 0.16 mmol/l each of dNTP, 0.5 μmol/l of each primer, and 1 unit of HotStarTaq™ DNA polymerase (Qiagen Inc., Valencia, CA, USA). The reaction mixture was initially denatured at 95°C for 15 min, followed by 35 cycles of 94°C for 30 s, 60°C for 30 s, and 72°C for 30 s. The PCR was completed by a final extension cycle at 72°C for 7 min. The PCR products were digested by the *Ngo*MIV and *Eco*RV restriction endonucleases for the Val-9Ala and Ile58Thr polymorphisms, respectively. The DNA fragments were then separated using 3% Nusieve/Agarose gel and visualized by ethidium bromide staining.

For the Val-9Ala polymorphism, the PCR product (107 bp) with C allele (Ala) was digested to two fragments (89 bp and 18 bp), whereas the PCR product with T allele (Val) cannot be cut by *Ngo*MIV. For the Ile58Thr polymorphism the PCR product (139 bp) with T allele (Ile) was digested to two fragments (117 bp and 22 bp), whereas the PCR product with C allele (Thr) cannot be cut by *Eco*RV. No Ile58Thr polymorphism was found in 400 individuals in our study, and we did not perform genotyping for this polymorphism in all participants.

The laboratory staff was blind to the identity of the participants. Quality control (QC) samples were included in the genotyping assays. Each 96-well plate contained one water, two CEPH 1347-02 DNA, two blinded QC DNA, and two unblinded QC DNA samples. The blinded and unblinded QC samples were taken from the second tube of study samples included in the study. The Val-9Ala genotypes determined for the blinded QC samples were in complete agreement with the genotypes determined for the study samples. Genotyping data were obtained from 1125 cases and 1197 control individuals. The major reasons for incomplete genotyping were insufficient DNA and unsuccessful PCR amplification.

### Statistic analysis

To evaluate case–control differences for categorical data, including genotype distribution and continuous variables, χ^2 ^test and t-test were used, respectively. Conditional logistic regression models adjusted for age were applied to estimate odds ratios (ORs) and 95% confidence intervals (CIs) to measure the strength of the association between the *MnSOD *gene Val-9Ala polymorphism and breast cancer risk [26,27]. Analyses stratified by menopausal status and age were conducted to check homogeneity of the association. Additional analyses stratified by body mass index (BMI), years of menstruation, and intake of fruits, vegetables, vitamin supplements, selenium, or antioxidant vitamins were conducted to evaluate the potential modifying effects of these variables on the association between the *MnSOD *genotypes and breast cancer risk. A composite dietary antioxidant index was derived to incorporate information on intake of four antioxidant nutrients (i.e. selenium and vitamins A, C, and E) [28]. Intake of each antioxidant nutrient was first standardized by subtracting the mean and dividing by the standard deviation, and then the antioxidant index was created by summing the standardized intake of these four antioxidant nutrients with equal weight [28]. All statistical tests were two-sided.

## Results

The distribution of selected demographic characteristics and major risk factors for breast cancer are shown in Table 1. Breast cancer cases and controls were similar in age and level of education. Elevated risks were observed for all known major breast cancer risk factors [29], including a prior history of breast fibroadenoma, physical inactivity, higher waist-to-hip ratio, higher BMI, early onset of menarche, late onset of menopause, and late age at first live birth. No apparent differences were found between individuals with genotyping data and those included in the whole study in any of the major known risk factors, demographic characteristics, and dietary antioxidant intake (data not shown), indicating that the chance of selection bias in this study is likely to be small.

The frequencies of the Ala allele were 14.3% and 14.0% in cases and control individuals, respectively. Genotype frequencies were 73.9% (Val/Val), 23.6 (Val/Ala), and 2.5% (Ala/Ala) for cases, and the respective frequencies were 73.9%, 24.2%, and 1.9% for control individuals (Table 2). The *MnSOD *Val-9Ala polymorphism was in Hardy–Weinberg equilibrium for both cases and control individuals. No Ile58Thr polymorphism was found in our study population. Thus, we report here the results for the Val-9Ala polymorphism.

Compared with women with the Val/Val genotype, breast cancer risk was slightly but not statistically significantly elevated for women with the Ala/Ala genotype (age-adjusted OR 1.3, 95% CI 0.7–2.3; Table 2). The Val/Ala genotype was unrelated to risk and was combined with the Val/Val genotype in some subsequent analyses. Additional adjustment for physical activity, BMI, waist-to-hip ratio, age at menarche, number of pregnancies, age at first birth, and family history of breast cancer had no appreciable effect on age-adjusted ORs, regardless of whether the analyses were done among all participants or stratified by menopausal status or age. Thus, only the age-adjusted ORs are presented. The elevated risk associated with the Ala/Ala genotype was restricted to premenopausal women (OR 1.8, 95% CI 0.9–3.7; for postmenopausal women OR 0.8, 95% CI 0.3–2.0) and women younger than 45 years (OR 1.8, 95% CI 0.7–4.3; for women older than 45 years OR 1.1, 95% CI 0.5–2.2).

Further analyses were conducted to evaluate the association of the *MnSOD *Val-9Ala polymorphism and breast cancer risk by duration (years) of menstruation and BMI, factors that are related to the duration and level of estrogen exposure (Table 3). The ORs for the Ala/Ala genotype were higher among premenopausal women with a higher BMI (OR 2.5, 95% CI 0.9–7.0) or a longer duration of menstruation (OR 2.6, 95% CI 0.8–8.0). Again, the ORs were not statistically significant. This increased risk was not observed in postmenopausal women (Table 3).

We further evaluated the association of the *MnSOD *Val-9Ala polymorphism with breast cancer risk by dietary antioxidant intake (Table 4). Intriguingly, the positive association with the Ala/Ala genotype among premenopausal women was consistently found to be stronger among those who had a low intake of fruits, vegetables, selenium, or antioxidant vitamins, including carotenes and vitamins A, C, and E, than among those who had a higher intake of these dietary factors. This pattern of association suggests a modifying effect, although neither the ORs nor the interaction tests were statistically significant, perhaps as a result of the small numbers of participants in the subgroups. The modifying effect of these antioxidant intakes on the association between the *MnSOD *Val-9Ala polymorphism and breast cancer risk was less apparent in the analyses among postmenopausal women. However, the postmenopausal case–control numbers were small. To illustrate the joint effects of dietary antioxidant intake, we further evaluated the association of the *MnSOD *Val-9Ala polymorphism with breast cancer risk by dietary antioxidant index. ORs for the Ala/Ala genotype were 2.4 (95% CI 0.6–9.5) and 2.2 (95% CI 0.5–9.4) among premenopausal and postmenopausal women, respectively, who had a lower dietary antioxidant index.

## Discussion

Ambrosone and colleagues previously reported that Val/Val genotype was significantly associated with an increased risk of breast cancer among premenopausal Caucasian women, particularly those who had a low intake of fruits and vegetables and of dietary ascorbic acid and α-tocopherol [17]. In this large-scale, population-based, case–control study, we found that breast cancer risk was slightly but not significantly elevated in Chinese women with the Ala/Ala genotype as compared with women with the Val/Val genotype, particularly among premenopausal women who had a low intake of fruits, vegetables, selenium, or antioxidant vitamins. A significant association of this polymorphism with breast cancer risk was not observed in present study, which might partly be due to low Ala allele frequency in Chinese women.

We observed in this study that women carrying the Ala/Ala genotype who had a higher BMI or a longer duration of menstruation were at higher risk of breast cancer, particularly among premenopausal women. Some ROS may be generated from estrogen metabolism through catechol redox cycling [3,4]. Mitrunen and coworkers [18] conducted a case–control study among 483 cases and 482 controls in a Finnish Caucasian population, and reported that the Ala allele was associated with breast cancer risk, with an OR of 1.5 in the Ala/Ala or Val/Ala groups compared with the Val/Val group. Postmenopausal women who had used estrogen replacement therapy and carried either the Ala/Ala or Val/Ala genotype had a 2.5-fold higher risk for breast cancer. Women who had used oral contraceptives and carried the Ala/Ala or Val/Ala genotype had a 3.0-fold higher risk for breast cancer [18]. More recently, Egan and coworkers [19] conducted a population-based case–control study among 476 cases and 502 controls in an American population. Overall, relative risks were not significantly elevated in women with one (OR 1.27, 95 CI 0.91–1.77) or two (OR 1.18, 95% CI 0.81–1.73) Ala alleles, as compared with the Val/Val genotype. Risk, however, was increased among premenopausal women carrying the Val/Ala genotype (OR 1.88), but not among women carrying the Ala/Ala genotype (OR 0.94) [19]. Women carrying the Ala/Ala or Val/Ala genotype who had used oral contraceptives or had higher BMI also had an increased risk for breast cancer [19]. Because of the low frequency of the Ala/Ala genotype and low percentage of estrogen use among Chinese women, we are unable to perform the analyses stratified by these exogenous estrogen exposures.

Several studies have evaluated the association of the *MnSOD *Val-9Ala polymorphism with other cancers, although the results were inconsistent among cancer sites. Recently, Woodson and coworkers [30] reported that the Ala/Ala genotype was associated with a 1.7-fold (95% CI 0.96–3.08) increased risk for prostate cancer as compared with the Val/Val genotype. No association of this polymorphism with colorectal adenomas was found in a sigmoidoscopy-based case–control study [31]. Recently, Lin and coworkers [32] reported no association of the *MnSOD *Val-9Ala polymorphism with lung cancer risk in a case–control study conducted in Taiwan. Interestingly, Wang and coworkers [33] reported that the Val allele was associated with lung cancer risk with ORs of 1.34 (95% CI 1.05–1.70) and 1.67 (95% CI 1.27–2.20) in the Val/Ala and Val/Val group, respectively. These differences in the associations of the *MnSOD *genotype with breast and lung cancers suggest that the role played by MnSOD in carcinogenesis may vary for different tumors. The mechanism underlying this tumor type difference remains to be investigated.

The SODs are the first and most important line of antioxidant enzyme defense against ROS and particularly O_2_^•- ^radical. It was predicted that *MnSOD *Val-9Ala polymorphism might alter transfer of the MnSOD enzyme into mitochondria [11,12]. Recently, Sutton and coworkers [13] reported that the Ala-MnSOD precursor generated 30–40% more of the active, matricial, and processed MnSOD homotetramer in mitochondrial matrix than did Val-MnSOD. Some human tumor cells lost MnSOD activity, and this loss has been shown to be responsible for at least part of the malignant phenotype [34,35]. MnSOD knockout mice exhibited increased oxidative DNA damage [36]. Over-expression of MnSOD in MCF-7 cell suppressed the malignant phenotype, as evidenced by decreased cell proliferation, clonogenic fraction in soft agar culture, and tumor growth in nude mice [37]. Recently, Soini and coworkers [38] reported that MnSOD expression is less frequent in the tumor cells of invasive breast carcinomas than in *in situ *carcinomas or non-neoplastic breast epithelial cells.

Three distinct isoforms of SOD have been identified in mammals. These three isoforms are encoded by three distinct genes located on different chromosomes [10]. All three *SOD *genes are polymorphic. It would be interesting to analyze jointly all of the three genes to evaluate any gene–gene interaction in relation to breast cancer risk. O_2_^•-^, if not scavenged by SOD, may react with nitric oxide radical (NO^•^) to form the strong oxidant peroxynitrite (ONOO^-^) [39]. MnSOD dismutates O_2_^•- ^to H_2_O_2_, which is further detoxified by GPX1 in mitochondria. If not be quenched, H_2_O_2 _will be converted to the more toxic hydroxyl radical (^•^OH). Thus, study of the joint effect of the *MnSOD*, nitric oxide synthase, and *GPX1 *gene polymorphisms might provide further information on the role played by oxidative stress in cancer risk.

Frequencies of the Val and Ala alleles of the *MnSOD *Val-9Ala polymorphism in our control population were 86.0% and 14.0%, respectively. The minor allele (Ala) frequency (14.0%) is comparable with that in Japanese (14.1%) [40] and Chinese (11.5%) [41] populations, but substantially lower than that in Caucasian populations [17-20]. The low frequency of the Ala allele in our study population contributes to a wider range of 95% CIs in OR estimations and limits the statistical power for stratified analyses. Small numbers of individuals, such as in the subgroups stratified by menopausal status in the present study, may result in unstable OR estimates. Studies with a larger sample size are needed to confirm the findings.

The current study has many strengths. First, the high participation rate and the population-based study design substantially reduce selection bias. Second, Chinese women living in Shanghai are relatively homogenous in their ethnic background; over 98% are from a single ethnic group (Han Chinese). Therefore, the potential confounding effect of ethnicity is not a major concern in this study. Third, the extensive information collected on lifestyle factors allowed comprehensive evaluation of their interaction with genetic polymorphisms. The risk estimates derived from age-adjusted and multivariable adjusted analyses were similar, indicating that a confounding effect is unlikely to be a concern in this study.

## Conclusion

In this population-based case–control study conducted in Chinese women, we found that the *MnSOD *Ala/Ala genotype was associated with a slightly but nonsignificantly elevated risk of breast cancer. The positive association was more evident among premenopausal women, particularly among those who consumed a low level of antioxidant vitamins or with high levels of oxidative stress. However, the study is limited by the low frequency of the Ala allele in the Chinese population, and most of the ORs were not statistically significant. Studies with a larger sample size are needed to confirm the findings.

## Author contributions

QC, X-OS, and WZ participated in interpretation of results and writing the manuscript. QC participated in laboratory assays. WW conducted the statistical analyses. J-RC, QD, and Y-TG participated in field operation.

## Competing interests

The authors declare that they have no competing interests.

## Abbreviations

BMI = body mass index; bp = base pairs; CI = confidence interval; GPX = glutathione peroxidase; MnSOD = manganese superoxide dismutase; OR = odds ratio; PCR = polymerase chain reaction; QC = quality control; ROS = reactive oxygen species.

## Figures and Tables

**Table 1:** Comparisons of cases and controls by selected demographic characteristics and major risk factors in the Shanghai Breast Cancer Study

Characteristic	Cases (n = 1125)	Controls (n = 1197)	P-value^a^
Demographic factors			
Age (years [mean ± SD])	47.63 ± 8.00	47.20 ± 8.78	0.217
Education, lower than middle school (%)	12.27	14.79	0.124
Major risk factors			
Breast cancer in first-degree relatives (%)	3.38	2.26	0.101
Ever had breast fibroadenoma (%)	9.78	5.26	< 0.001
Age at menarche (years [mean ± SD])	14.41 ± 1.64	14.71 ± 1.73	0.004
Age at first live birth^b ^(years [mean ± SD])	26.82 ± 4.07	26.17 ± 3.83	< 0.001
Age at menopause^c ^(years [mean ± SD])	48.17 ± 4.65	47.46 ± 4.95	0.036
Physically active in past 10 years (%)	19.40	26.00	< 0.001
Body mass index (mean ± SD)	23.54 ± 3.39	23.25 ± 3.45	0.047
Waist-to-hip ratio (mean ± SD)	0.81 ± 0.06	0.80 ± 0.06	0.004

^a^From χ^2 ^test (categorical variables) or T test (continuous variables). ^b^Among parous women.

^c^Among postmenopausal women. SD, standard deviation.

**Table 2:** Association between the manganese superoxide dismutase polymorphism and breast cancer risk in the Shanghai Breast Cancer Study

Genotype	Cases (*n *[%])	Controls (*n *[%])	OR^a^	95% CI
**All subjects**				
Val/Val	831 (73.9)	884 (73.9)	1.0	Reference
Val/Ala	266 (23.6)	290 (24.2)	1.0	0.8–1.2
Ala/Ala	28 (2.5)	23 (1.9)	1.3	0.7–2.3
Val/Val and Val/Ala	1097 (97.5)	1174 (98.1)	1.0	Reference
Ala/Ala	28 (2.5)	23 (1.9)	1.3	0.7–2.3
**Stratified analyses by menopausal status**				
Premenopausal women				
Val/Val and Val/Ala	729 (97.2)	751 (98.4)	1.0	Reference
Ala/Ala	21 (2.8)	12 (1.6)	1.8	0.9–3.7
Postmenopausal women				
Val/Val and Val/Ala	363 (98.1)	419 (97.4)	1.0	Reference
Ala/Ala	7 (1.9)	11 (2.6)	0.8	0.3–2.0
			*P *for interaction = 0.147	
**Stratified analyses by age**				
Age < 45 years				
Val/Val and Val/Ala	442 (97.1)	488 (98.4)	1.0	Reference
Ala/Ala	13 (2.9)	8 (1.6)	1.8	0.7–4.3
Age ≥ 45 years				
Val/Val and Val/Ala	655 (97.8)	686 (97.9)	1.0	Reference
Ala/Ala	15 (2.2)	15 (2.1)	1.1	0.5–2.2
			*P *for interaction = 0.380	

^a^Adjusted for age. CI, confidence interval; OR, odds ratio.

**Table 3:** Associations of breast cancer with the manganese superoxide dismutase polymorphism, stratified by lifestyle factors, in the Shanghai Breast Cancer Study

Stratified variable (by median)	MnSOD genotypes					
	
	Val/Val		Val/Ala		Ala/Ala	
			
	*n*^a^	OR^b ^(95% CI)	*n*^a^	OR^b ^(95% CI)	*n*^a^	OR^b ^(95% CI)
**Premenopausal women**						
BMI						
≤ Median	250/275	1.0 (ref.)	73/99	0.9 (0.6–1.2)	8/7	1.3 (0.5–3.7)
> Median	300/288	1.0 (ref.)	106/89	1.1 (0.8–1.6)	13/5	2.5 (0.9–7.0)
Years of menstruation^c^						
≤ Median	203/286	1.0 (ref.)	77/91	1.2 (0.8–1.7)	8/8	1.4 (0.5–3.7)
> Median	347/277	1.0 (ref.)	102/97	0.8 (0.6–1.2)	13/4	2.6 (0.8–8.0)
**Postmenopausal women**						
BMI						
≤ Median	126/156	1.0 (ref.)	39/52	1.0 (0.6–1.6)	4/7	0.7 (0.2–2.5)
> Median	152/161	1.0 (ref.)	46/50	1.0 (0.6–1.6)	3/4	0.8 (0.2–3.7)
Years of menstruation^c^						
≤ Median	128/172	1.0 (ref.)	44/54	1.1 (0.7–1.8)	2/6	0.4 (0.1–2.2)
> Median	150/145	1.0 (ref.)	41/48	0.8 (0.5–1.3)	5/5	1.0 (0.3–3.5)

^a^Number of cases/controls. ^b^Adjusted for age. ^c^Years of menstruation = menopausal age or age at interview for premenopausal women – menarche age. CI, confidence interval; MnSOD, manganese superoxide dismutase; OR, odds ratio; ref., reference.

**Table 4:** Associations of breast cancer with the manganese superoxide dismutase polymorphism polymorphism, stratified by food and nutrient intake, in the Shanghai Breast Cancer Study

Stratified variable	Premenopausal women						Postmenopausal women					
	
	Val/Val		Val/Ala		Ala/Ala		Val/Val		Val/Ala		Ala/Ala	
						
	*n*^a^	OR^b ^(95% CI)	*n*^a^	OR^b^ (95% CI)	*n*^a^	OR^b ^ (95% CI)	*n*^a^	OR^b ^(95% CI)	*n*^a^	OR^b^ (95% CI)	*n*^a^	OR^b ^ (95% CI)
Total vegetables												
≤ Median	273/290	1.0 (ref.)	89/88	1.1 (0.8–1.6)	9/4	2.4 (0.7–8.0)	145/164	1.0 (ref.)	49/44	1.3 (0.8–2.0)	4/6	0.7 (0.2–2.7)
> Median	277/273	1.0 (ref.)	90/100	0.9 (0.6–1.2)	12/8	1.5 (0.6–3.7)	133/153	1.0 (ref.)	36/58	0.7 (0.5–1.2)	3/5	0.8 (0.2–3.3)
Total fruits												
≤ Median	260/281	1.0 (ref.)	95/98	1.1 (0.8–1.5)	7/3	2.6 (0.7–10.0)	152/166	1.0 (ref.)	38/44	1.0 (0.6–1.6)	4/5	1.0 (0.3–3.8)
> Median	290/282	1.0 (ref.)	84/90	0.9 (0.6–1.2)	14/9	1.5 (0.7–3.6)	126/151	1.0 (ref.)	47/58	1.0 (0.6–1.5)	3/6	0.6 (0.1–2.3)
Vitamin A												
≤ Median	238/286	1.0 (ref.)	86/92	1.1 (0.8–1.6)	10/4	2.9 (0.9–9.2)	111/165	1.0 (ref.)	29/46	1.0 (0.6–1.6)	5/4	1.9 (0.5–7.3)
> Median	312/277	1.0 (ref.)	93/96	0.9 (0.6–1.2)	11/8	1.3 (0.5–3.3)	167/152	1.0 (ref.)	55/56	0.9 (0.6–1.4)	2/7	0.3 (0.1–1.3)
Carotene												
≤ Median	281/287	1.0 (ref.)	88/90	1.0 (0.7–1.5)	10/4	2.6 (0.8–8.4)	131/157	1.0 (ref.)	41/52	0.9 (0.6–1.5)	3/6	0.6 (0.1–2.4)
> Median	269/276	1.0 (ref.)	91/98	0.9 (0.7–1.3)	11/8	1.4 (0.5–3.5)	147/160	1.0 (ref.)	44/50	1.0 (0.6–1.6)	4/5	0.9 (0.2–3.4)
Vitamin E												
≤ Median	288/282	1.0 (ref.)	96/95	1.0 (0.7–1.4)	10/5	1.9 (0.7–5.7)	156/155	1.0 (ref.)	48/53	0.9 (0.6–1.5)	4/7	0.6 (0.2–2.1)
> Median	262/281	1.0 (ref.)	83/93	1.0 (0.7–1.4)	11/7	1.8 (0.7–4.9)	122/162	1.0 (ref.)	37/49	1.1 (0.6–1.7)	3/4	1.0 (0.2–4.8)
Vitamin C												
≤ Median	275/282	1.0 (ref.)	92/96	1.0 (0.7–1.4)	12/4	3.1 (1.0–9.8)	137/161	1.0 (ref.)	43/48	1.1 (0.7–1.7)	5/6	1.0 (0.3–3.3)
> Median	275/281	1.0 (ref.)	87/92	1.0 (0.7–1.3)	9/8	1.1 (0.4–3.0)	141/156	1.0 (ref.)	42/54	0.9 (0.6–1.4)	2/5	0.5 (0.1–2.5)
Selenium												
≤ Median	256/278	1.0 (ref.)	78/101	0.9 (0.6–1.2)	6/3	2.1 (0.5–8.6)	115/164	1.0 (ref.)	37/48	1.1 (0.7–1.8)	5/3	2.3 (0.5–9.9)
> Median	294/285	1.0 (ref.)	101/87	1.1 (0.8–1.6)	15/9	1.7 (0.7–3.9)	163/153	1.0 (ref.)	48/54	0.9 (0.5–1.3)	2/8	0.3 (0.1–1.2)
Vitamin supplement												
No	488/480	1.0 (ref.)	162/174	0.9 (0.7–1.2)	20/10	1.9 (0.9–4.2)	227/255	1.0 (ref.)	70/80	1.0 (0.7–1.5)	5/10	0.6 (0.2–1.7)
Yes	62/83	1.0 (ref.)	17/14	1.6 (0.7–3.6)	1/2	0.7 (0.1–7.8)	51/62	1.0 (ref.)	15/22	0.9 (0.4–2.0)	2/1	2.1 (0.2–24.3)
Dietary antioxidant index^c^												
≤ Median	262/280	1.0 (ref.)	89/98	1.0 (0.7–1.4)	7/3	2.4 (0.6–9.5)	126/161	1.0 (ref.)	43/51	1.1 (0.7–1.8)	5/3	2.2 (0.5–9.4)
> Median	288/283	1.0 (ref.)	90/90	1.0 (0.7–1.4)	14/9	1.6 (0.7–3.8)	152/156	1.0 (ref.)	42/51	0.9 (0.5–1.4)	2/8	0.3 (0.1–1.2)

^a^Number of cases/controls. ^b^Adjusted for age. ^c^Dietary antioxidant index was derived as described in the Methods section. CI, confidence interval; OR, odds ratio; ref., reference.
